# Serum positive thyroglobulin antibodies: an old problem with new questions

**DOI:** 10.1590/2359-3997000000263

**Published:** 2017-04-01

**Authors:** Fernanda Vaisman

**Affiliations:** 1 Instituto Nacional de Câncer Universidade Federal do Rio de Janeiro Rio de Janeiro RJ Brasil Instituto Nacional de Câncer (Inca). Programa de Pós-Graduação Stricto-Sensu em Endocrinologia, Universidade Federal do Rio de Janeiro (UFRJ), Rio de Janeiro, RJ, Brasil

## INTRODUCTION

Serum thyroglobulin (Tg) is a cornerstone on the follow up of differentiated thyroid cancer (DTC) and is widely used for diagnostic and prognostic purposes (1). Some factors might interfere in the measurement, being the most common the presence of thyroglobulin antibodies (TgAB) (2). This is known to happen in nearly one third of patients. In this scenario, image modalities become even more important during follow up as serum Tg measurement in not reliable and TgAB absolute values and/or TgAB’s curve over time is not as accurate. Furthermore, it is also known that lymph node metastasis can be found in more than 50% of patients at diagnosis and it is the most common site of persistence/recurrence of disease (1). The appropriate initial surgical procedure can lower the frequency of persistence/recurrence of lymph node metastatic disease.

In addition to a well-performed neck ultrasound, the ultrasound guided fine needle aspiration biopsy (FNAB) should be performed in every suspicious lymph node in order to confirm malignancy. As these biopsies still carry up to 10% of false negative results (3) recommended to measure Tg levels in needle washouts (Tg-FNAB). Therefore, another issue came up during the last couple of years: does the presence of serum TgAB interfere with the measurement of Tg-FNAB as they are present also in the washouts?

## METHODS

Martins-Costa and cols. (4), assessed 232 FNAB samples obtained from suspicious lymph neck nodes from 144 DTC patients. Regular cytology, Tg-FNAB, TgAB in the washouts and also serum Tg and TgAB were performed in all patients. They have used the literature-validated cutoffs. Tg-FNAB was performed using a commercially available assay with functional sensitivity of 1.0 ng/mL and if Tg-FNAB was lower than 10 ng/mL and patient had serum Tg AB positivity, another assay with lower sensitivity was used to exclude contamination by TgAB.

## RESULTS

Patients were divided into two groups: group 1 (203 FNAB samples): serum negative TgAB and group 2 (29 FNAB samples): serum positive TgAB. In the first group, all patients with Tg-FNAB > 10 ng/mL had metastatic disease confirm by histology or ^I13^1 uptake, however, only 57% had positive cytology. When Tg-FNAB was < 10 ng/mL, only 10% had confirm metastatic disease and FNAB was able to identify only 2%. In group 2, when Tg-FNAB was above 10 ng/mL, also all patients had confirmed metastatic disease and positive cytology but when less than 10 ng/mL, around 45% had confirmed metastatic disease by histology or ^I13^1 uptake but only 25% had positive cytology. In both groups there were a high frequency of non-diagnostic cytology FNABs when Tg-FNAB was less than 10 ng/mL, showing that neither Tg-FNAB nor cytology were able to identify those cases of lymph node metastasis.

## COMMENTS

The authors nicely showed that Tg-FNAB is a useful tool to diagnose lymph node metastasis as they proved that, when above 10 ng/mL, all patients had confirmed metastatic disease, independently on the Tg-AB serum status. Thus, if Tg-FNAB is high in a suspicious lymph node, is likely that will be confirmed as metastatic disease. Interestingly, Tg-FNAB was even more accurate than cytology. That information reinforces the recommendation that Tg-FNAB should be measured when suspicious lymph nodes are biopsied in order to increase sensitivity.

Conversely, when Tg-FNAB was lower, the accuracy was not as good but neither was cytology.

Even more important, in the present study, the authors also showed that the same phenomenon occurs in serum TgAB positive and negative patients, suggesting that the presence of TgAB in the serum does not interfere significantly in Tg-FNAB measurement. This is an ongoing discussion in the literature. Shin and cols., studied 239 lymph nodes from 201 PTC patients and found that the presence of serum TgAB in fact interfered with Tg-FNAB measurement (5) and similarly, Jo and cols. suggested that serum TgAB might lower Tg-FNAB. This last study proposed to lower the Tg-FNAB cutoff for serum TgAb positive patients (6). However, those studies performed FNAB of suspicious lymph nodes before thyroid surgery when serum TgAB levels were higher than in Martins-Costa’s and cols. study. Therefore, the interference of serum TgAB in Tg-FNAB measurement might be significant when serum TgAB are higher and differences in cutoffs for both measurements should be considered in those situations.
